# Understanding the Role of Exercise and Probiotic Interventions on Non-Alcoholic Fatty Liver Disease Alleviation in Zebrafish: Dialogue Between the Gut and Liver

**DOI:** 10.3390/ijms26031360

**Published:** 2025-02-06

**Authors:** Xueyan Gu, Liyan Yuan, Long Gan, Zehui Zhang, Shibiao Zhou, Zhenliang Fu, Yu Liu, Zaijun Xin, Shaohang Cheng, Xinyou Zhou, Hualong Yan, Qiyu Wang

**Affiliations:** 1 Physical Education College, Jiangxi Normal University, Nanchang 330022, China; guxueyan1122@126.com (X.G.); yuanliyan66@163.com (L.Y.); zhoushibiao11@163.com (S.Z.); fuzhenliang527@163.com (Z.F.); chengshaohang99@126.com (S.C.); zhouxinyou0603@163.com (X.Z.); yanhualong123@126.com (H.Y.); 2 Energy Research Institute, Jiangxi Academy of Sciences, Nanchang 330012, China; ganlong0730@126.com; 3 School of Water Resources & Environmental Engineering, East China University of Technology, Nanchang 330013, China; zzh02190220@163.com; 4 Research Institute of Microbiology, Jiangxi Academy of Sciences, Nanchang 330012, China; liuyu@jxas.ac.cn (Y.L.); zaijunxin@126.com (Z.X.)

**Keywords:** Non-alcoholic fatty liver disease, exercise, probiotics, gut microbiota, zebrafish

## Abstract

Non-alcoholic fatty liver disease (NAFLD), the most prevalent chronic liver illness, is characterized by hepatic steatosis. Exercise and probiotics can regulate the gut microbiota to treat NAFLD; however, their combined effects and the mechanisms of gut-liver communication remain unclear. Inconsistent results on probiotic efficacy further warrant investigation. In this study, zebrafish fed a high-fat diet (HFD) for six weeks were subjected to swimming exercise (HFDE), probiotic intervention (HFDP), or a combination of both (HFDEP) for 10 weeks to explore their effects on NAFLD and the corresponding mechanism. The results showed that NAFLD alleviation followed the order HFDEP > HFDE > HFDP. HFDEP and HFDE treatments effectively reduced Body Mass Index (BMI), relative liver weight, liver vacuolation density, lipid droplets in liver sections, triglyceride, free fatty acid, glucose, and pyruvic acid. In contrast, a single probiotic treatment had limited impact, suggesting a complementary role in NAFLD treatment. Glucose and fatty acid metabolism were central to the “gut–liver” axis. The reduced conversion of glucose to pyruvic acid, decreased fatty acid synthesis and esterification, and accelerated fatty acid transformation to CO_2_ contributed to NAFLD improvement under HFDE and HFDEP treatments. This study provides promising theoretical groundwork for potential prevention and treatment strategies for NAFLD.

## 1. Introduction

Non-alcoholic fatty liver disease (NAFLD) is a common cause of chronic liver disease worldwide, defined by the presence of steatosis in more than 5% of hepatocytes with little or no alcohol consumption [1]. NAFLD is prevalent in both high-income and low-income countries. NAFLD is prevalent in 6–35% of the global population, with 90 million people in the United States suffering from NAFLD, accounting for 30% globally [2,3]. NAFLD can progress from the more benign condition of non-alcoholic fatty liver (NAFL) to non-alcoholic steatohepatitis (NASH), which is at the more severe end of the spectrum [4,5]. NASH increases the risk of liver disease and possibly non-liver-related outcomes, including cirrhosis, liver failure, and hepatocellular carcinoma, as well as non-liver-associated adverse outcomes related to increased cardiovascular disease and malignancy [6]. The public health problems associated with NAFLD should not be ignored.

Currently, lifestyle change through exercise is one major method for NAFLD treatment [7]. The clinical practice guidelines of the European Association for the Study of Liver (EASL), the European Association for the Study of Obesity (EASO), and the American Association for the Study of Liver Diseases (AASLD) state that exercise triggers a 5–10% weight loss in overweight/obese NAFLD patients [8,9]. The National Institute for Health and Care Excellence (NICE) guidelines also recommends exercise as the primary treatment method for NAFLD [10]. It was not only suitable for humans but also suitable for animals. A 12-week high-intensity interval training program reduced the blood glucose content and waist circumference in NAFLD patients [11]. In a high-fat diet (HFD)-induced NAFLD mouse model, exercise training significantly decreased hepatic steatosis and fibrosis [12]. Exercise intervention is an effective method of manipulating NAFLD progression.

Microbiome evolution is essential for the pathogenesis of NAFLD development [6,13]. Human studies have identified several consistent microbiome signatures discriminating healthy individuals from those with NAFLD, and animal studies have demonstrated the potential causal role of the gut microbiota in NAFLD [14,15]. The “gut–liver” axis between the intestinal microbiota and the host explains the potential link to liver damage. Evidence shows an interaction between exercise, gut microbiota, and NAFLD [16,17]. The effects of exercise in rugby players were compared to sedentary overweight and obese controls, and a significantly more diverse gut microbiota and lower levels of inflammatory and metabolic markers were observed in highly active rugby players [18]. In HFD-induced male C57BL/6 mice, treadmill exercise significantly ameliorated hepatic function and mitigated lipid accumulation; *Dubosiella* mostly mediated these benefits [19]. Regulating bile acids in the “gut–liver” axis through exercise is another treatment strategy for NAFLD [20]. These facts indicate that exercise can improve NAFLD by regulating the gut microbiota.

Probiotics are also common intestinal microecological preparations used to improve NAFLD by restoring the gut microbial balance. In a placebo-controlled trial, an oral combination of *Lactobacillus bulgaricus* and *Streptococcus thermophilus* improved the liver aminotransferase levels in NAFLD patients [21]. The supplementation of *Saccharomyces boulardii* powder for 12 weeks in NAFLD patients significantly decreased the levels of total bilirubin (TBil), aspartate aminotransferase (AST), alanine aminotransferase (ALT), γ-glutamyl transpeptidase (GGT), serum total cholesterol (T-CHO), low-density lipoprotein cholesterol (LDL-C), and fasting insulin [22]. In mice, administration of the potential probiotic *Bacteroides thetaiotaomicron* ameliorated NAFLD by decreasing the *Firmicutes*/*Bacteroidetes* ratio, enhancing gut–liver folate and unsaturated fatty acid metabolism [23]. However, other studies reported contradictory results. A randomized, double-blind, placebo-controlled trial showed that the oral administration of a multi-strain probiotic containing six different *Lactobacillus* and *Bifidobacterium* species at 30 billion CFU/d for 6 months had no significant effect on hepatic steatosis [24]. Therefore, further evidence is required to verify the therapeutic effects of probiotics on NAFLD.

Considering the individual mitigating effects of exercise and probiotics on NAFLD, their combined effect on NAFLD has been explored in some studies. In tetracycline-induced NAFLD rats, high-intensity interval training combined with *Lactobacillus rhamnosus Gorbach-Goldin (LGG)* supplementation minimized cell destruction and inflammation in the liver tissue due to NAFLD by improving TIMP-1, MMP-2, and LIPA expression [25]. A meta-analysis of randomized clinical trials conducted up to April 2024 demonstrated that probiotic supplementation combined with exercise training elicited better results than exercise alone in terms of body weight (BW), AST, ALT, GGT, triglyceride (TG), T-CHO, LDL, and homeostatic model assessment for insulin resistance (HOMA-IR) in patients with NAFLD [26]. However, current studies on the combined effects of exercise and probiotics on NAFLD progression are limited, especially in different species, and their mechanisms of action remain unclear.

Therefore, it is necessary to investigate the single and combined effects of exercise and probiotics on NAFLD progression and to elucidate the corresponding mechanism of action. Zebrafish are excellent models for establishing NAFLD because of their high homology to the human genome. In this study, we fed male zebrafish that were 3 months old with a HFD for 6 weeks to establish a zebrafish NAFLD model and then treated the NAFLD zebrafish by single and joint interventions of exercise and probiotics. The single and joint effects of exercise and probiotics on NAFLD progression and corresponding mechanisms involved with “gut–liver” axis were then determined by monitoring the hepatic developmental indices, bio-indicators related to glucose and fatty acid metabolism, as well as gut microbial alteration. This will be an important supplement in the field of NAFLD manipulation.

## 2. Results

### 2.1. HFD Induced NAFLD by Acting on Glucose and Fatty Acid Metabolism

**Figure 1:**
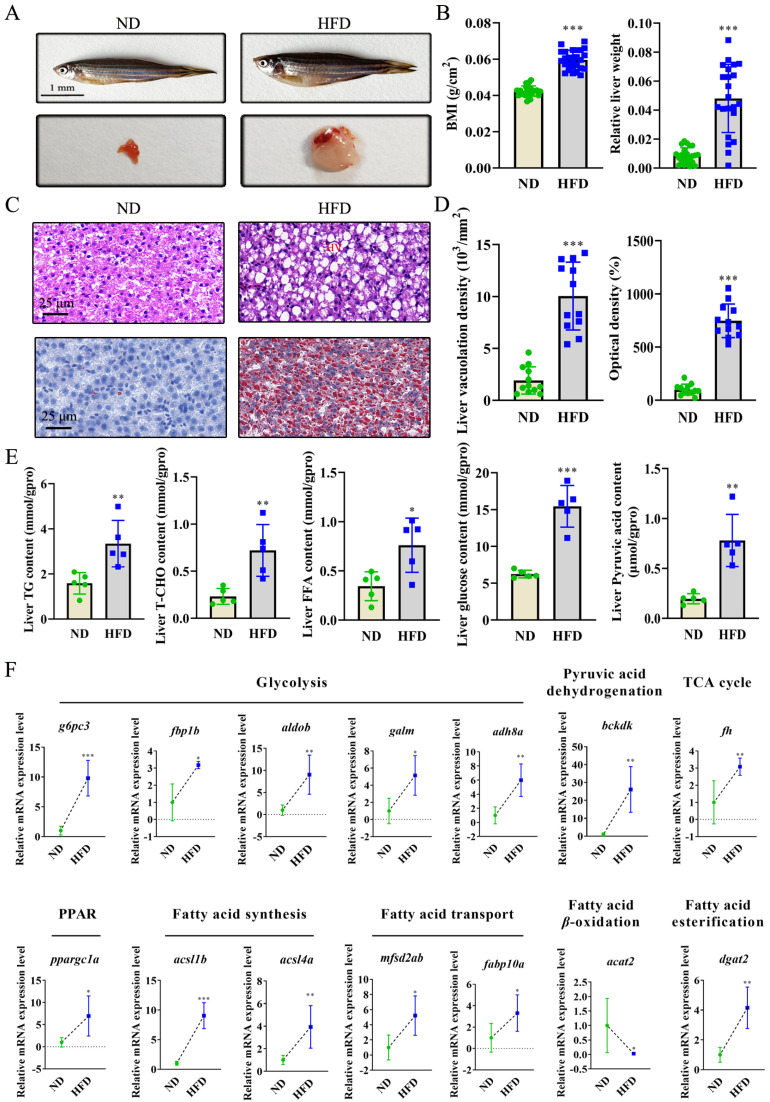
High-fat diet (HFD)-induced non-alcoholic fatty liver disease (NAFLD) progression involved in glucose and fatty acid metabolism: (**A**,**B**) Photographed fish and liver and corresponding Body Mass Index (BMI) and relative liver weight in normal diet (ND) and HFD groups. (**C**,**D**) Liver sections by hematoxylin-eosin (HE) and Oil Red O (ORO) staining and their corresponding liver vacuolation density in HE-stained sections and optical density in ORO staining. (**E**) Biochemical determination on lipid indicators including triglyceride (TG), total cholesterol (T-CHO), free fatty acid (FFA), glucose, and its metabolic intermediate of pyruvic acid. (**F**) Transcriptional levels of the genes related to glucose metabolism (glycolysis, pyruvic acid dehydrogenation, TCA cycle), and fatty acid metabolism (PPAR pathway transduction, fatty acid synthesis, fatty acid transport, fatty acid esterification, and fatty acid β-oxidation), determined using qRT-PCR. Values are the mean ± SD. * *p* < 0.05, ** *p* < 0.01, *** *p* < 0.001 vs. control.

After 16 weeks of treatment, HFD intervention significantly induced NAFLD, with the presence of obese fish and fatty livers (Figure 1A), in which the Body Mass Index (BMI) and relative liver weight increased dramatically by approximately 0.41- and 4.54- fold (Figure 1B). Further histopathological sections examined through hematoxylin-eosin (HE) and Oil Red O (ORO) staining showed that liver vacuolation density was remarkably upregulated and lipid droplets accumulated, with increases of 423.91% and 647.08%, respectively (Figure 1C,D). Biochemical analysis showed that TG and T-CHO levels increased in the HFD group (Figure 1E). TG synthesis-related indicators, including free fatty acids (FFA), glucose, and its metabolic intermediate pyruvic acid, were also determined, showing that HFD treatment increased the contents of FFA, glucose, and pyruvic acid (Figure 1E). The expression profile of genes related to glucose and fatty acid metabolism demonstrated that HFD treatment promoted glycolysis, pyruvic acid dehydrogenation, the tricarboxylic acid (TCA) cycle, peroxisome proliferator-activated receptor (PPAR) pathway transduction, fatty acid synthesis, fatty acid transport, and fatty acid esterification and inhibited fatty acid β-oxidation (Figure 1F).

### 2.2. Altered Gut Microbiota May Contribute to HFD-Induced NAFLD

**Figure 2:**
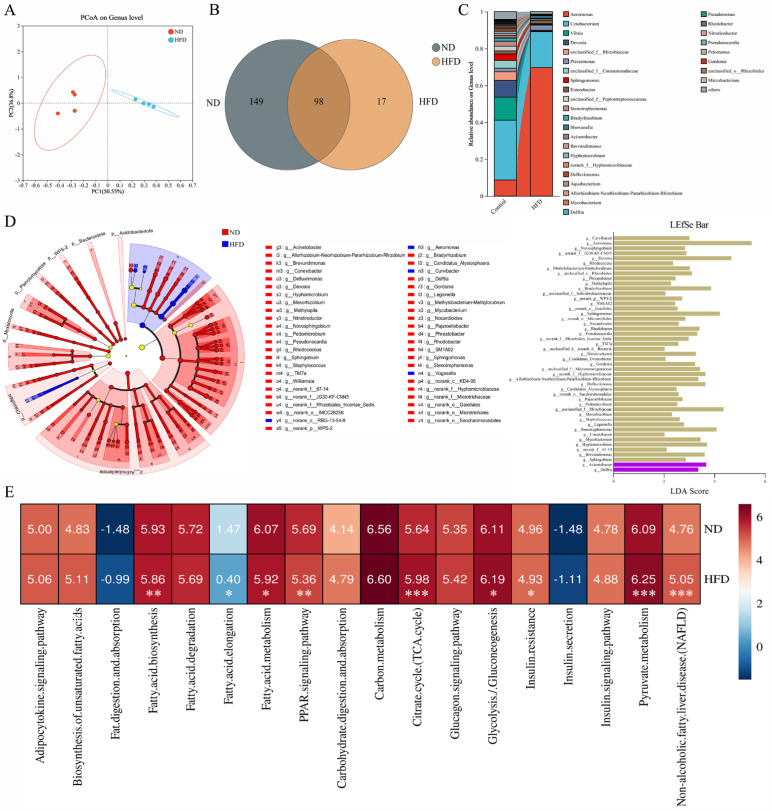
Alterations in gut microbiota may induce NAFLD by changing the glucose and fatty acid metabolism: (**A**) Principal coordinate analysis (PCoA) of gut microbiota on the genus level in different groups. (**B**) Venn chart for genera in ND and HFD groups. (**C**) Percentage of community abundance on the genus level after ND and HFD treatments. (**D**) Linear discriminant analysis (LDA) effect size (LEfSe) of the gut microbiota from the phylum to genus levels with LDA values >2 and *p* values <0.05 in different groups. The yellow nodes represent microbial groups that do not differ significantly between groups, or have no significant effect on differences between groups. (**E**) Comparison of biological processes associated with glucose and fatty acid metabolism in ND and HFD groups based on log10 of KEGG pathway abundances at level 3 using the phylogenetic investigation of communities by the reconstruction of unobserved states 2 (PICRUSt2). * *p* < 0.05, ** *p* < 0.01, *** *p* < 0.001 vs. control.

The altered microbiome is an essential pathogen in NAFLD progression, and gut microbial changes were evaluated by 16S rRNA gene sequencing. Principal coordinate analysis (PCoA) indicated a significant difference between the normal diet (ND) and HFD groups (Figure 2A). HFD treatment significantly decreased the number of genera from 247 in the ND group to 115 in the HFD group (Figure 2B), and the abundance of each genus changed (Figure 2C). Linear discriminant analysis (LDA) effect size (LEfSe) showed that 48 genera contributed to the significant deviation between the ND and HFD groups (Figure 2D). The functional abundance of glucose and fatty acid metabolic pathways was assessed using the phylogenetic investigation of communities by the reconstruction of unobserved states 2 (PICRUSt2), which showed that gut microbial changes in the HFD group affected fatty acid biosynthesis, fatty acid elongation, fatty acid metabolism, the PPAR signaling pathway, the TCA cycle, glycolysis/gluconeogenesis, insulin resistance, pyruvate metabolism, and NAFLD progression (Figure 2E and Table S1).

### 2.3. Single and Joint Effects of Exercise and Probiotics on NAFLD Progression

**Figure 3:**
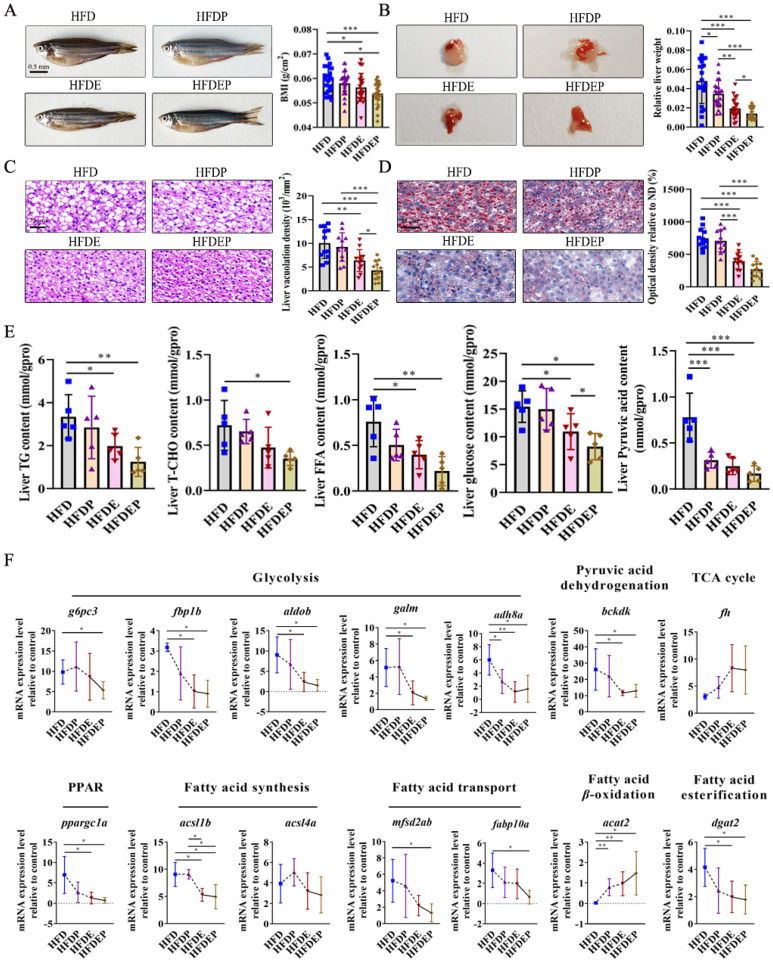
Effects of different intervention strategies on NAFLD: (**A**) Photographed fish and corresponding BMI. (**B**) Photographed liver and corresponding relative liver weight. (**C**,**D**) Histopathological sections of liver by HE and ORO staining, as well as their corresponding liver vacuolation density and optical density. Scale bar: 25 µm. (**E**) Determination on lipid indicators. (**F**) Expression levels of genes related to glucose (glycolysis, pyruvic acid dehydrogenation, and TCA cycle) and fatty acid metabolism (PPAR pathway transduction, fatty acid synthesis, fatty acid transport, fatty acid esterification, and fatty acid β-oxidation). Values are the mean ± SD. * *p* < 0.05, ** *p* < 0.01, *** *p* < 0.001 vs. control.

Considering the mitigating effects of exercise and probiotics on NAFLD, their combined effects on NAFLD were explored. The BMI was significantly decreased in the HFD with exercise (HFDE) and HFD with exercise and probiotics (HFDEP) groups compared to that in the HFD group, and there was no change after a single probiotic intervention (Figure 3A). Regarding the relative liver weight, the HFD with probiotics (HFDP), HFDE, and HEDEP treatments all decreased the relative liver weight in comparison with the HFD group (Figure 3B). Further histopathological examination showed that the liver vacuolation density determined by HE staining and optical density determined by ORO staining were both decreased in the HFDE and HEDEP groups (Figure 3C,D). The alleviation effect on NAFLD progression followed the order HFDEP > HFDE > HFDP, in which the HFDP did not mitigate NAFLD (Figure 3A–D). The biochemical analysis of lipid indicators showed that the HFDEP decreased the levels of hepatic TG, T-CHO, FFA, glucose, and pyruvic acid, whereas the HFDE decreased the levels of TG, FFA, glucose, and pyruvic acid (Figure 3E). Additionally, pyruvic acid levels decreased in the HFDP group (Figure 3E). The quantification of gene expression related to glucose and fatty acid metabolism demonstrated that the HFDEP inhibited glycolysis, pyruvic acid dehydrogenation, PPAR pathway transduction, fatty acid synthesis, fatty acid transport, and fatty acid esterification and promoted fatty acid β-oxidation (Figure 3F). The promotion of fatty acid β-oxidation and the inhibition of glycolysis, pyruvic acid dehydrogenation, PPAR pathway transduction, fatty acid synthesis, and fatty acid esterification were also observed in the HFDE group (Figure 3F). Regarding the HFDP group, only glycolysis and fatty acid β-oxidation were impacted, with the down-regulation of *adh8a* and up-regulation of *acat2* (Figure 3F).

### 2.4. Single and Joint Effects of Exercise and Probiotics on Gut Microbiota Composition

**Figure 4:**
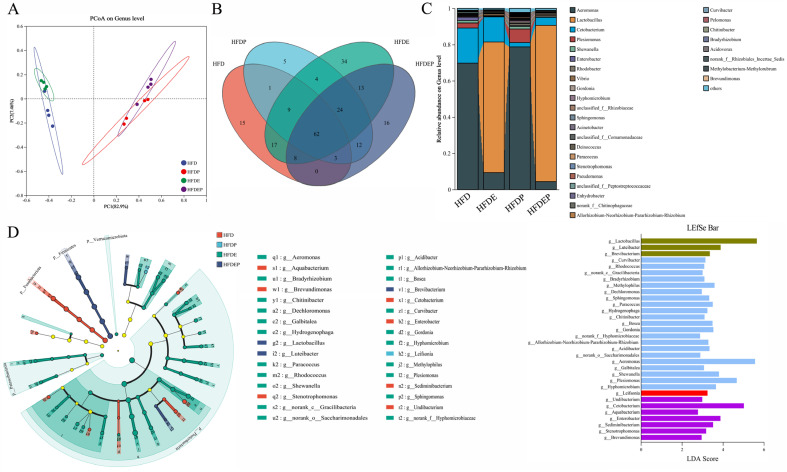
Gut microbial change under different intervention modes: (**A**) PCoA of gut microbiota on the genus level in different groups. Venn chart (**B**) and percentage of community abundance (**C**) for gut genera after different treatments. (**D**) LEfSe of the gut microbiota from the phylum to genus levels with LDA values > 2 and *p* values < 0.05 in different groups. The yellow nodes represent microbial groups that do not differ significantly among groups, or have no significant effect on differences among groups.

PCoA revealed significant differences at the genus level between HFD/HFDE and HFDP/HFDEP groups (Figure 4A). In total, 115, 120, 171, and 138 genera were identified in the HFD, HFDP, HFDE, and HFDEP groups, respectively (Figure 4B). Single and joint interventions by the probiotic *Lactobacillus rhamnosus GG* supplementation and exercise altered the composition and the level of the gut microbial community in the genus in comparison with the HFD group, and the gut microbiota in the HFDP and HFDEP groups showed a similar pattern (Figure 4C). LEfSe showed that 27 genera contributed to the significant deviation between the HFD and HFDP/HFDE/HFDEP groups, including 1 genus in the HFDP group, 21 genera in the HFDE group, and 3 genera in the HFDEP group (Figure 4D).

### 2.5. Gut Microbial Functional Change by Single and Joint Interventions of Exercise and Probiotics

**Figure 5:**
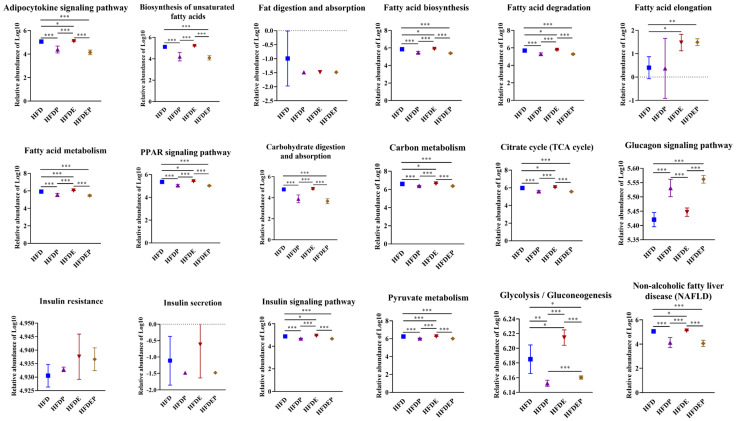
Gut microbial functional change by different intervention modes. Comparison of biological processes associated with glucose and fatty acid metabolism in different groups based on log10 of KEGG pathway abundances at level 3 using PICRUSt2. Values are the mean ± SD. * *p* < 0.05, ** *p* < 0.01, *** *p* < 0.001 vs. control.

PICRUSt2 analysis indicated that these significantly altered genera in different groups all affected the processes of the adipocytokine signaling pathway, the biosynthesis of unsaturated fatty acids, fatty acid biosynthesis, fatty acid degradation, fatty acid metabolism, PPAR signaling pathway, carbohydrate digestion and absorption, carbon metabolism, the TCA cycle, glucagon signaling pathway, glycolysis/gluconeogenesis, the insulin signaling pathway, pyruvate metabolism, and NAFLD progression to different extents (Figure 5 and Table S2). Among the affected processes, the biosynthesis of unsaturated fatty acids, carbohydrate digestion and absorption, and the glucagon signaling pathway were not altered in the HFDE group in comparison with the HFD group but were changed in HFDP and HFDEP groups (Figure 5 and Table S2). Fatty acid elongation was only changed in HFDE and HFDEP groups compared with the HFD group (Figure 5 and Table S2).

### 2.6. Lipid Indicators Change Associated with Altered Gut Microbiota

**Figure 6:**
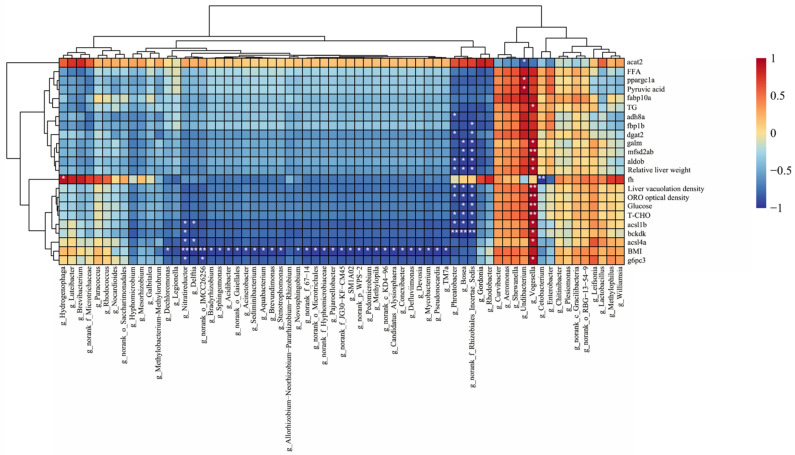
Correlation analysis between altered gut microbiota and lipid indicators. * *p* < 0.05, and ** *p* < 0.01.

To verify whether the identified altered gut microbiota were associated with hepatic lipid metabolic disorders via glucose and fatty acid metabolism, correlation analysis between the altered microbiota at the genus level and lipid metabolism-related indicators was performed (Figure 6). The results showed that *g_Vogesella* was correlated with all lipid indicators except BMI, *dgat2*, *fabp1b*, *adh8a*, *fabp10a*, pyruvic acid, *ppargc1a*, FFA, and *acat2*, whereas pyruvic acid, *ppargc1a*, and *acat2* were correlated only with *g_Undibacterium*. The genera *g_Bosea*, *g_Phreatobacter*, and *g_norank_f_Rhizobiales_Incertae_Sedis* demonstrated a similar significant correlation pattern, in which *g_Bosea* and *g_norank_f_Rhizobiales_Incertae_Sedis* were both correlated with *bakdk*, *acsl1b*, T-CHO, glucose, ORO optical density, liver vacuolation density, relative liver weight, *aldob*, *mfsd2ab*, and *galm*, and *g_norank_f_Rhizobiales_Incertae_Sedis* was also correlated with *dgat2* and *fbp1b*. The genus *g_Phreatobacter* correlated with *bakdk*, *acsl1b*, T-CHO, ORO optical density, liver vacuolation density, relative liver weight, *aldob*, *dgat2*, and *adh8a*. BMI correlated with 31 genera in total. Additionally, *g_Nitratireductor* was associated with *g6pc3*, *acsl4a*, *bckdk*, *acsl1b*, *g_Delftia* was associated with *acsl4a*, *acsl1b*, and *g_norank_o_IMCC26256* was associated with *g6pc3*. The gene *fh* was only associated with *g_Cetobacterium*.

## 3. Discussion

NAFLD involves excessive lipid deposition in the liver and is often accompanied by obesity, diabetes, and other metabolic disorders [27]. Gut microbiota has been increasingly recognized as a pivotal determinant of NAFLD [13,28,29,30]. Compelling evidence has revealed that intervention with exercise and probiotics can mitigate NAFLD by changing the gut microbiota [17,31]; however, research on the joint effects of exercise and probiotics on NAFLD progression is still limited, and their mechanism of action remains unknown. Additionally, the therapeutic effect of probiotics on NAFLD must be further confirmed because of inconsistent results [24]. Based on this research gap, this study used the zebrafish NAFLD model to explore the single and joint effects of exercise and probiotics on NAFLD progression and the corresponding mechanism.

HFD intervention successfully induced NAFLD in zebrafish, which was specifically manifested as an increase in BMI, relative liver weight, liver vacuolation density, lipid droplets in liver sections, and TG and T-CHO contents (Figure 1A–E). In view of the previously reported link between gut microbiota changes and NAFLD progression in animals and humans [14,15], changes in the intestinal flora and their possible mediated functions after HFD induction were profiled. The 48 genera that contributed to the significant deviation between the ND and HFD groups may have affected fatty acid metabolism (fatty acid biosynthesis, fatty acid elongation, fatty acid metabolism, and PPAR signaling pathway) and glucose metabolism (TCA cycle, glycolysis/gluconeogenesis, insulin resistance, and pyruvate metabolism) to induce NAFLD (Figure 2D,E). Both fatty acids and glucose metabolism participate in TG metabolism [32]. Fatty acid metabolism-related pathways, including fatty acid biosynthesis, fatty acid transport, and fatty acid β-oxidation, are regulated by the PPAR pathway [33]. The synthesized fatty acids are finally transformed into TG through fatty acid esterification [34]. Fatty acids can also be metabolized into CO_2_ through fatty acid β-oxidation [33]. In glucose metabolism, glucose can be converted into pyruvic acid through glycolysis and then transformed into acetyl-CoA through pyruvic acid dehydrogenation, in which acetyl-CoA can not only be synthesized into fatty acids but can also be metabolized into CO_2_ through the TCA cycle [32,35]. These results suggest that HFD-induced NAFLD may be attributable to fatty acid and glucose metabolism changes through the “gut–liver” axis, which affects hepatic TG synthesis.

To verify this hypothesis, the levels of FFAs, glucose, and pyruvic acid in the liver and the expression of genes related to fatty acid and glucose metabolism were quantified. Regarding fatty acid metabolism, HFD treatment increased the FFA content in the liver and promoted PPAR pathway transduction, fatty acid synthesis, fatty acid transport, and fatty acid esterification (Figure 1E,F). Conversely, fatty acid β-oxidation was inhibited (Figure 1F). This illustrates that more fatty acids accumulated in the liver, and excess fatty acids were transformed into TG to induce NAFLD. The inhibition of fatty acid β-oxidation further impeded the transformation of fatty acids into CO_2_. Regarding glucose metabolism, HFD intervention upregulated the contents of glucose and its metabolite pyruvic acid and promoted glycolysis, pyruvic acid dehydrogenation, and the TCA cycle (Figure 1E,F), which indicated that more glucose was transformed into pyruvic acid, burdening hepatic TG degradation. The TCA cycle is activated in a compensatory manner to reduce hepatic TG accumulation. The enhanced TCA cycle during NAFLD progression was also observed in previous studies [36,37]. Our biochemical analysis preliminarily revealed a mechanistic link between gut microbiota alterations and NAFLD occurrence. Previous studies have demonstrated the mechanism of the “gut–liver” axis during NAFLD progression, deeming that gut microbiota generate a variety of bioactive substances, including lipopolysaccharides, peptidoglycan, DNA, and extracellular vesicles, as well as the metabolites that interact with the host liver cells through the portal vein, which have been associated with the glycolipid metabolism in the liver [13,28,29,30]. This study further elucidated the biological processes regulated by gut microbes. The detailed mechanistic link provided the basis for analyzing single and combined effects and the corresponding mechanisms of exercise and probiotics on NAFLD alleviation.

The individual and combined effects of exercise and probiotics on NAFLD mitigation were explored. Overall, the alleviation effect on NAFLD followed the order of HFDEP > HFDE > HFDP, in which the HFDEP and HFDE both effectively alleviated NAFLD progression, which was presented by a decrease in BMI, relative liver weight, liver vacuolation density, lipid droplets in liver sections, TG, FFA, glucose, and pyruvic acid (Figure 3A–E). However, the HFDP group did not improve NAFLD and only decreased the relative liver weight and pyruvic acid content, and the levels of other lipid indicators did not change (Figure 3A–E). Combined treatment with exercise and probiotics has been verified to improve NAFLD in humans and rats. A systematic review and meta-analysis of randomized clinical trials showed that probiotic supplementation combined with exercise training elicited better results than exercise alone on liver enzymes AST and GGT, lipid profiles of LDL-C and T-CHO, and insulin resistance in patients with NAFLD [26]. In rats with tetracycline-induced NAFLD, interval exercise with probiotic *LGG* supplementation minimizes cell destruction and inflammation in the liver tissue due to NAFLD by improving the gene expression profiles [25]. This study further confirmed the beneficial effects of joint interventions of exercise and probiotics on NAFLD improvement in another species. No consistent conclusion has been reached regarding the treatment of NAFLD using probiotics alone. Although numerous studies have demonstrated that probiotics are ideal oral preparations for ameliorating NAFLD, some studies elucidated the opposite results. In a randomized, double-blind, placebo-controlled trial involving ultrasound-diagnosed NAFLD patients who were supplemented with either a probiotic sachet (MCP^®^ BCMC^®^ strains) or a placebo for 6 months, no significant changes in hepatic steatosis and fibrosis levels were found at the end of the study [24]. In another randomized, double-blinded, placebo-controlled trial with 35 patients who were administered two sachets of VSL#3^®^ probiotic supplement or a placebo, no significant differences were observed in the biomarkers of liver injury [38]. The present study also found that probiotic treatment alone had a weak effect on NAFLD. Therefore, probiotics may play a complementary role in the treatment of NAFLD. Further studies with larger sample sizes, longer durations, and different probiotic strains are required to evaluate the benefits of probiotics in NAFLD treatment.

Considering the decrease in the contents of hepatic TG, FFA, glucose, and pyruvic acid after HFDE and HFDEP treatments in comparison with the HFD group (Figure 3E) and the effect on NAFLD by the change in the fatty acid and glucose metabolism through the “gut–liver” axis, the expression of genes related to fatty acid and glucose metabolism were also quantified in the HFDP, HFDE, and HFDEP groups. The inhibition of glycolysis, pyruvic acid dehydrogenation, the PPAR pathway transduction, fatty acid synthesis, fatty acid transport, and the fatty acid esterification and promotion of fatty acid β-oxidation occurred in the HFDE and HFDEP groups (Figure 3F). This indicated that the HFDE and HFDEP groups reduced the transformation efficiency of glucose to pyruvic acid and the accumulation of fatty acids in the liver. The resulting reduced amounts of pyruvic acid and fatty acids and inhibited fatty acid esterification further impeded TG formation. Fatty acid β-oxidation activation simultaneously accelerated the conversion from fatty acids into CO_2_, decreasing the TG content. The superior effect of HFDEP treatment compared with that of HFDE treatment demonstrated a more effective improvement in NAFLD. Regarding HFDP treatment, only *adh8a* and *acat2* expressions changed, namely, which were involved in glycolysis and fatty acid β-oxidation, respectively (Figure 3F). The slightly altered expression profiles of the genes related to glucose and fatty acid metabolism in the HFDP group had a weak effect on NAFLD improvement. Previous studies have described the positive effects of exercise combined with probiotic administration on the alleviation of NAFLD in terms of hepatic function, insulin resistance, cell destruction, and hepatic inflammation [25,26]. This study innovatively explained why the effects on NAFLD improvement followed the order HFDEP > HFDE > HFDP from the glucose and fatty acid metabolism perspective.

Coincidentally, the intestinal flora changes in the HFDP, HFDE, and HFDEP groups also affected the molecular processes of glucose and fatty acid metabolism, including the adipocytokine signaling pathway, biosynthesis of unsaturated fatty acid, fatty acid biosynthesis, fatty acid degradation, fatty acid elongation, fatty acid metabolism, carbon metabolism, the TCA cycle, the glucagon signaling pathway, glycolysis/gluconeogenesis, and the insulin signaling pathway (Figure 4D and Figure 5), which further suggested that exercise and probiotic interventions improved NAFLD by acting on glucose and fatty acid metabolism in the “gut–liver” axis. Given that the affected processes in the HFDP and HFDEP groups showed a similar pattern but the HFDP group showed a very weak effect on NAFLD mitigation, probiotics may have a complementary role in treating NAFLD. To verify the crosstalk between the gut and liver after intervention with exercise and probiotics to treat NAFLD, a correlation analysis between the altered microbiota and lipid indicators was performed (Figure 6). The significant correlation verified the existence of a “gut–liver” axis during NAFLD treatment through exercise and probiotics, and glucose and fatty acid metabolism participated in the communication between the gut and liver.

In conclusion, this study investigated the single and joint effects of exercise and probiotics on NAFLD alleviation in zebrafish for the first time and found that treatment with exercise in combination with probiotics had the best effect, followed by exercise alone. Probiotic treatment alone resulted in a slight improvement in NAFLD progression; however, this was not significant, indicating a complementary role in treating NAFLD. Mechanically, glucose and fatty acid metabolism are important dialogue links in the “gut–liver” axis. Inhibition of the conversion of glucose to pyruvic acid and fatty acid synthesis as well as the acceleration of the transformation of fatty acids to CO_2_ contributed to NAFLD alleviation after exercise and exercise combined with probiotic treatments.

## 4. Materials and Methods

### 4.1. NAFLD Zebrafish Model

**Figure 7:**
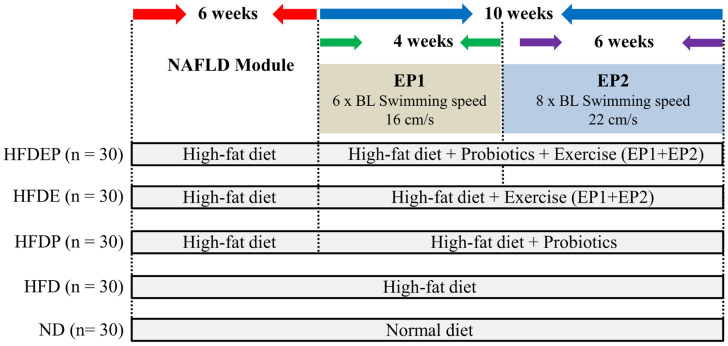
Schematic of experimental design.

A total of 220 3-month-old male wild-type adult zebrafish (AB strain) were purchased from the China Zebrafish Resource Center. They were maintained in a zebrafish aquarium facility (Tecniplast Zebtec, Tecniplast, Buguggiate, Italy) under a 14 h light/10 h dark photoperiod at 27 ± 1 °C. Of these, 155 fish were induced with NAFLD in the first 6 weeks via a HFD of 0.1 g/day of egg yolk and 0.25 g/day of fresh hatched *Artemia* per fish (Figure 7). The fish were fed three times daily. In weeks 0, 1, 2, 4, 6 of NAFLD inducement, the fish and livers were photographed; body length, body weight, and liver weight were recorded to calculate their BMI (*n* = 5 per time point) and relative liver weight (*n* = 5 per time point). Where BMI = body weight/body length^2^, and relative liver weight = liver weight/body weight. Liver samples were collected simultaneously, in which five (*n* = 5) were obtained at each time point for TG and T-CHO determination, and 10 liver samples were subjected to (HE) staining (*n* = 5) and ORO staining (*n* = 5) after 6 weeks of induction to verify the establishment of NAFLD.

### 4.2. Experimental Design

After the successful induction of NAFLD (Figure S1), the remaining 120 HFD fish were treated under four methods for 10 weeks: a HFD, a HFD with probiotic *Lactobacillus rhamnosus GG* (HFDP), a HFD with exercise (HFDE), and a HFD with exercise and probiotics (HFDEP). The HFD group continued to be fed the HFD. In the HFDP treatment group, the HFD fish were fed the lyophilized probiotic *Lactobacillus rhamnosus GG* (10^8^ cells/g; Culturelle, San Francisco, CA, USA). For HFDE treatment, HFD fish were subjected to swimming exercises, as previously described [39,40]. Briefly, they were placed in a swimming tunnel that compelled them to swim at a 6 × BL/s swimming speed (16 cm/s) for the first four weeks, which was then increased to 8 BL/s (22 cm/s) for the next six weeks. During the exercise period, the fish were first acclimated for 30 min in a swimming tunnel and then exercised for 5 d per week for 4 h per day. In the HFDEP group, HFD fish were subjected to both swimming exercises and probiotics. The normal diet (ND) group was fed fresh hatched *Artemia* at 0.06 g/day per fish for 16 weeks. A schematic of the experimental design is shown in Figure 7.

### 4.3. Sample Preparation and Collection

After treatment, the fish and livers were photographed, and body length, body weight, and liver weight were recorded to calculate the BMI (*n* = 26 per group) and relative liver weight (*n* = 22 per group). Liver samples were also collected for histopathological, biochemical, and gene expression analyses, in which four samples (*n* = 4, one fish per sample) from each group were subjected to HE staining, four samples (*n* = 4, one fish per sample) from each group underwent ORO staining, five samples (*n* = 5, two fish per sample) from each group underwent biochemical determination, and five samples (*n* = 5, one fish per sample) from each group underwent gene expression analysis. Fecal samples (*n* = 4, 5 fish per sample) were obtained after treatment for 16S rRNA sequencing.

All experimental procedures involving animals were approved by the Independent Animal Care and Use Committee of Jiangxi Academy of Sciences (Approval No. 2022–012).

### 4.4. Hepatic Histopathological Examination

The collected liver samples from each group were fixed in 4% paraformaldehyde overnight for histopathological examination. The livers were sliced into 3–4 µm thick samples before undergoing HE, and the frozen liver sections of 8–10 µm thickness were applied for ORO staining. HE and ORO staining were performed as described previously [41,42]. Slice images were captured using a light microscope (Olympus IX 53, TYO, Japan). Using ImageJ V.1.8.0 software, the hepatic vacuolation density and optical density features in ORO staining were blind-scored based on three fields of view per sample (https://imagej.net/ij/index.html (accessed on 13 March 2024)).

### 4.5. Hepatic Biochemical Determination

Zebrafish liver samples acquired for biochemical analysis were homogenized in 10 volumes of ice-cold phosphate-buffer saline (50 mM, pH 7.0) using an electric homogenizer. The homogenates were centrifuged at 6000× *g* for 10 min at 4 °C. Supernatants were collected to determine the levels of TG, T-CHO, FFA, glucose, and pyruvic acid using commercial kits (Nanjing Jiancheng Bioengineering Institute, Nanjing, China) following the manufacturer’s instructions.

### 4.6. Gene Expression Analysis

Total RNA was extracted from the collected livers using an Ultrapure RNA Kit (CWBIO, Beijing, China). The cDNA synthesis and real-time polymerase chain reaction (qRT-PCR) were performed as described previously [32]. The gene-specific primer sequences involved in glycolysis (*g6pc3*, *fbp1b*, *aldob*, *galm*, *adh8a*), pyruvic acid dehydrogenation (*bckdk*), PPAR (*ppargc1a*), fatty acid synthesis (*acsl1b*, *acsl4a*), fatty acid β-oxidation (*acat2*), fatty acid transport (*mfsd2ab*, *fabp10a*), the tricarboxylic acid (TCA) cycle (*fh*), and fatty acid esterification (*dgat2*) are listed in Table S3. *β-actin* was selected as the housekeeping gene for normalization. The fold-change in the target gene expression levels was calculated using the 2^−△△Ct^ method.

### 4.7. 16S rRNA Gene Sequencing

Fecal samples were subjected to 16S rRNA gene sequencing to explore the changes in the gut microbiota. Total microbial genomic DNA was extracted using the E.Z.N.A.^®^ Soil DNA Kit (Omega Bio-tek, Norcross, GA, USA). The hypervariable region V3-V4 of the bacterial 16S rRNA gene were amplified with primer pairs 338F (5′-ACTCCTACGGGAGGCAGCAG-3′) and 806R (5′-GGACTACHVGGGTWTCTAAT-3′). Purified amplicons were pooled in equimolar amounts and pair-end sequenced on an Illumina MiSeq PE300 platform (Illumina, San Diego, CA, USA) according to the standard protocols of Majorbio Bio-Pharm Technology Co. Ltd. (Shanghai, China), followed by analysis in QIME (version 1.8.0) to reveal variations in the gut microbiota.

### 4.8. Bioinformatic Analysis

Bioinformatic analysis of the gut microbiota was performed using the Majorbio Cloud platform (https://cloud.majorbio.com (accessed on 13 September 2024)). The similarity among the microbial communities in the different groups was determined by principal coordinate analysis (PCoA) based on Bray–Curtis dissimilarity using the Vegan v2.5-3 package. Linear discriminant analysis (LDA) effect size (LEfSe) (http://huttenhower.sph.harvard.edu/LEfSe (accessed on 17 June 2024)) was used to identify the most abundant genera of bacteria among the different groups (LDA score > 2, *p* < 0.05). Metagenomic function based on the KEGG pathway at level 3 was predicted using PICRUSt2. Pearson’s correlation analysis between the abundance of the altered microbiota at the genus level and the levels of lipid indicators was performed using the OmicStudio platform (https://www.omicstudio.cn/tool (accessed on 5 November 2024)).

### 4.9. Statistical Analysis

All data are presented as the means ± the standard deviation of the mean (SD). Statistical analyses were conducted using the GraphPad Prism 9.0 software (GraphPad Software Inc., La Jolla, CA, USA). Significant differences among different groups were determined using the one-way analysis of variance, followed by Duncan’s test (≥3 groups) or an unpaired *t*-test (two independent groups) with a statistical significance threshold of *p* < 0.05.

## Data Availability

The datasets generated and/or analyzed in the present study are available from the corresponding author upon reasonable request.

## Supplementary Materials

The following supporting information can be downloaded at https://www.mdpi.com/article/10.3390/ijms26031360/s1.
