# Supplementary material for: Understanding the Role of Exercise and Probiotic Interventions on Non-Alcoholic Fatty Liver Disease Alleviation in Zebrafish: Dialogue Between the Gut and Liver
Source: Int J Mol Sci. 2025 Feb 6;26(3):1360. doi: 10.3390/ijms26031360 (PMC11818905; doi:10.3390/ijms26031360)

Table S1. Abundances of KEGG pathway related to glucose and fatty acid metabolism by log10 in ND and HFD groups.

	ND_1	ND_2	ND_3	ND_4	HFD_1	HFD_2	HFD_3	HFD_4
Adipocytokine signaling pathway	4.923278	5.093215	4.933562	5.058013	5.058683	5.090255	5.066598	5.038198
Biosynthesis of unsaturated fatty acids	4.408012	5.148025	4.639365	5.10503	5.135998	5.183889	5.118331	5.007765
Carbohydrate digestion and absorption	3.409512	4.92796	3.778151	4.446801	4.818628	4.846071	4.786631	4.699144
Carbon metabolism	6.464261	6.64918	6.481264	6.635512	6.609248	6.628211	6.60102	6.562697
Citrate cycle (TCA cycle)	5.662217	5.527987	5.74079	5.621088	5.995709	6.030681	5.981576	5.90708
Fat digestion and absorption	-1.48149	-1.48149	-1.48149	-1.48149	-1.48149	-1.48149	0.477121	-1.48149
Fatty acid biosynthesis	5.908367	5.969634	5.914045	5.943363	5.858573	5.87462	5.861949	5.841341
Fatty acid degradation	5.531155	5.832897	5.593591	5.942326	5.69861	5.734187	5.703698	5.625389
Fatty acid elongation	0.90309	1.31513	1.514149	2.154333	0	0.69897	0.90309	0
Fatty acid metabolism	6.003746	6.117389	5.996033	6.148114	5.893784	5.918318	5.899582	5.95945
Glucagon signaling pathway	5.266801	5.483421	5.28197	5.384861	5.430842	5.445129	5.419609	5.386393

Glycolysis / Gluconeogenesis	6.05874	6.181548	6.057605	6.123774	6.192883	6.204423	6.184307	6.158567
Insulin resistance	4.941412	4.964465	4.94312	4.974758	4.933801	4.934067	4.928724	4.925443
Insulin secretion	-1.48149	-1.48149	-1.48149	-1.48149	0	-1.48149	-1.48149	-1.48149
Insulin signaling pathway	4.672347	4.871638	4.722045	4.841727	4.891944	4.914584	4.873573	4.829277
Non-alcoholic fatty liver disease (NAFLD)	4.639716	4.834718	4.763926	4.790304	5.011097	5.077961	5.018811	5.094204
PPAR signaling pathway	5.696948	5.724442	5.829637	5.513438	5.355285	5.392396	5.367087	5.309894
Pyruvate metabolism	6.07283	6.0709	6.08577	6.142755	6.234848	6.25036	6.224534	6.286453

Table S2. Abundances of KEGG pathway related to glucose and fatty acid metabolism by log10 in HFDP, HFDE, and HFDEP groups.

	HFD_1	HFD_2	HFD_3	HFD_4	HFDP_1	HFDP_2	HFDP_3	HFDP_4	HFDE_1	HFDE_2	HFDE_3	HFDE_4	HFDEP_1	HFDEP_2	HFDEP_3	HFDEP_4
Glucagon signaling pathway	5.430842	5.445129	5.419609	5.386393	5.555357	5.505402	5.504622	5.559503	5.464014	5.451777	5.429343	5.44159	5.569629	5.54244	5.572256	5.563291
Glycolysis / Gluconeogenesis	6.192883	6.204423	6.184307	6.158567	6.156403	6.151557	6.146868	6.154823	6.221083	6.210458	6.224862	6.201685	6.161429	6.15716	6.161256	6.160921
Insulin resistance	4.933801	4.934067	4.928724	4.925443	4.932263	4.933691	4.933402	4.931911	4.935233	4.929078	4.936796	4.949069	4.93408	4.932989	4.936702	4.942497
Insulin secretion	0	-1.48149	-1.48149	-1.48149	-1.48149	-1.48149	-1.48149	-1.48149	0.477121	-1.48149	-1.48149	0	-1.48149	-1.48149	-1.48149	-1.48149

Insulin signaling	4.89	4.91	4.87	4.82	4.65	4.70	4.71	4.64	4.92	4.93	4.92	4.94	4.651	4.678	4.647	4.685
pathway	1944	4584	3573	9277	5316	93	1095	508	5638	9593	7176	412	513	232	427	571
Carbohydrate																
digestion and	4.81	4.84	4.78	4.69	3.66	4.20	4.19	3.47	4.89	4.88	4.74	4.79	3.550	3.946	3.425	3.737
absorption	8628	6071	6631	9144	2096	4635	672	3195	3879	2581	2662	7129	595	796	534	935
	6.60	6.62	6.60	6.56	6.35	6.42	6.43	6.34	6.64	6.62	6.63	6.63	6.349	6.383	6.348	6.388
Carbon metabolism	9248	8211	102	2697	6319	4723	5005	6303	4151	7729	3879	5348	341	23	795	31
Citrate cycle (TCA	5.99	6.03	5.98	5.90	5.54	5.66	5.69	5.51	6.05	6.03	6.04	6.06	5.534	5.600	5.531	5.606
cycle)	5709	0681	1576	708	1091	9518	1482	693	4839	2605	3022	8466	007	299	478	871
Pantothenate and CoA	5.86	5.87	5.84	5.81	5.53	5.64	5.64	5.52	5.88	5.86	5.87	5.87	5.520	5.576	5.515	5.564
biosynthesis	0797	343	8682	9396	9155	0191	414	3868	292	7938	7024	128	825	273	277	09
	6.23	6.25	6.22	6.28	6.00	6.06	6.06	5.99	6.26	6.25	6.24	6.25	5.996	6.025	5.996	6.022
Pyruvate metabolism	4848	036	4534	6453	2085	0591	7352	3257	7492	2508	0989	1244	972	507	279	677

Adipocytokine	5.05	5.09	5.06	5.03	4.18	4.64	4.64	4.09	5.10	5.08	5.10	5.08	4.007	4.388	3.969	4.206
signaling pathway	8683	0255	6598	8198	5514	3037	2909	3603	0411	7095	898	1277	364	918	851	16
PPAR signaling	5.35	5.39	5.36	5.30	4.99	5.11	5.14	4.98	5.41	5.39	5.40	5.42	4.989	5.049	4.999	5.073
pathway	5285	2396	7087	9894	6652	5665	3786	1587	4031	0869	305	2767	83	302	811	833
Biosynthesis of																
unsaturated fatty	5.13	5.18	5.11	5.00	3.98	4.51	4.54	3.74	5.21	5.19	5.18	5.17	3.930	4.300	3.898	4.215
acids	5998	3889	8331	7765	0911	3802	9817	9807	8392	2767	9932	2407	288	21	059	765
Ether lipid	3.62	3.73	3.66	3.28	2.27	3.26	3.67	2.92	3.54	3.19	4.17	3.81	2.647	2.824	3.206	3.783
metabolism	0127	7175	6683	3579	6714	2135	0627	8396	3432	76	4718	4235	197	438	408	878
Fat digestion and	-1.4	-1.4	0.47	-1.4	-1.4	-1.4	-1.4	-1.4	-1.4	-1.4	-1.4	-1.4	-1.48	-1.48	-1.48	-1.48
absorption	8149	8149	7121	8149	8149	8149	8149	8149	8149	8149	8149	8149	149	149	149	149
Fatty acid	5.85	5.87	5.86	5.84	5.39	5.58	5.59	5.36	5.88	5.87	5.88	5.88	5.342	5.460	5.340	5.440
biosynthesis	8573	462	1949	1341	0017	7352	5675	6202	4755	275	7867	5133	454	69	197	583

	5.69	5.73	5.70	5.62	5.20	5.38	5.44	5.18	5.76	5.73	5.75	5.80	5.209	5.292	5.243	5.379
Fatty acid degradation	861	4187	3698	5389	6278	283	2174	4724	1738	0327	3377	8479	115	303	227	257
		0.69	0.90		-1.4	0.90	1.46	0.60	1.38	1.39	1.14	1.97	1.431	1.322	1.591	1.633
Fatty acid elongation	0	897	309	0	8149	309	2398	206	6142	794	6128	7724	364	219	065	468
	5.89	5.91	5.89	5.95	5.43	5.67	5.70	5.40	6.03	6.01	6.03	6.04	5.386	5.530	5.398	5.543
Fatty acid metabolism	3784	8318	9582	945	263	3166	4199	1555	439	5407	5005	831	841	131	254	245
Non-alcoholic fatty	5.01	5.07	5.01	5.09	3.86	4.41	4.53	3.68	5.11	5.08	5.11	5.11	3.816	4.184	3.862	4.359
liver disease (NAFLD)	1097	7961	8811	4204	5302	6799	8276	9532	5663	25	8202	181	352	711	748	374

Table S3. The primer sequences for qRT-PCR.

Gene name	Accession NO.	Forward Primer	Reversed Primer
β-actin	NM_131031.2	CGAGCAGGAGATGGGAACC	CAACGGAAACGCTCATTGC
acsl1b	NM_001003569	CGATTGCGGAGCTGGCCTGTTAC	GCCGGTGAGTTTTGCCCATGTTCT
acsl4a	NM_200649	TCGCTCCACGGATCGCTTCG	GCTGCCAGTCCGCTCCCAAAA
mfsd2ab	NM_001003570	TGCGCGATCTATGTTGTCTGTGCTG	GCGGAGAGCATAATGACGAGCAAGA
fabp10a	NM_152960	CAGCGGGACGTGGCAGGTTTAC	TCGCTGCCGTTCTGCTGGATTTC
acat2	NM_131370	CCGTGGGGCTCAAGATCAACTGG	TTGCTGCCATGCCGAGGAAACT
g6pc3	NM_001080066	GGGGGTGTTTCTGAAGCGCTCTGT	CAGCGTCACGGTTAAGGGAGGAAAA
fbp1b	NM_213132	TGGAGGGCGCTATGTGGGGTCTA	ACCTCCAGCCTGCTCCATGATGAAG
aldob	NM_194367	GCCGTAGCTTTCGCGACCTTCTCTT	TGGGCACAGCGTTCAGACAGACC
galm	NM_001002373	TGCGGTCAGCGAGTGTCAGTGTG	ACCCGGCCTACAACAGCTCCAAAG
adh8a	NM_001001946	GCTCGGCTGCGGCATCACTACT	CAGCCAGACCGACAGCACCGAGT

ppargc1a	XM_017357139	CCCCCCTTCGTCTTCCTCACTGG	CCCCGCTATAGAAGGCTTGCTGAGG
bckdk	NM_213060	GCCCGCTTTCCCTTCATTCCACT	AGGGCCGCTGTTGGTCATGCTATC
dgat2	NM_001030196	CCATACTTGCTGCATATTCC	ATGTCATGATAAACTGCAGC
fh	NM_200963	GCGGGTACGTGCAGCAGGTGAA	GCCGCCAGAGCCTCGAACTTATTG

Figure S1. NAFLD model establishment. Photographed fish (A), liver (B), and liver sections by HE (C) and ORO (D) staining before and after HFD inducement. (E) Quantification of BMI, relative liver weight, hepatic TG, and T-CHO in ND and HFD treatment groups. Values are the mean ± SD. * $p < 0.05$, ** $p < 0.01$, *** $p < 0.001$ vs. control.

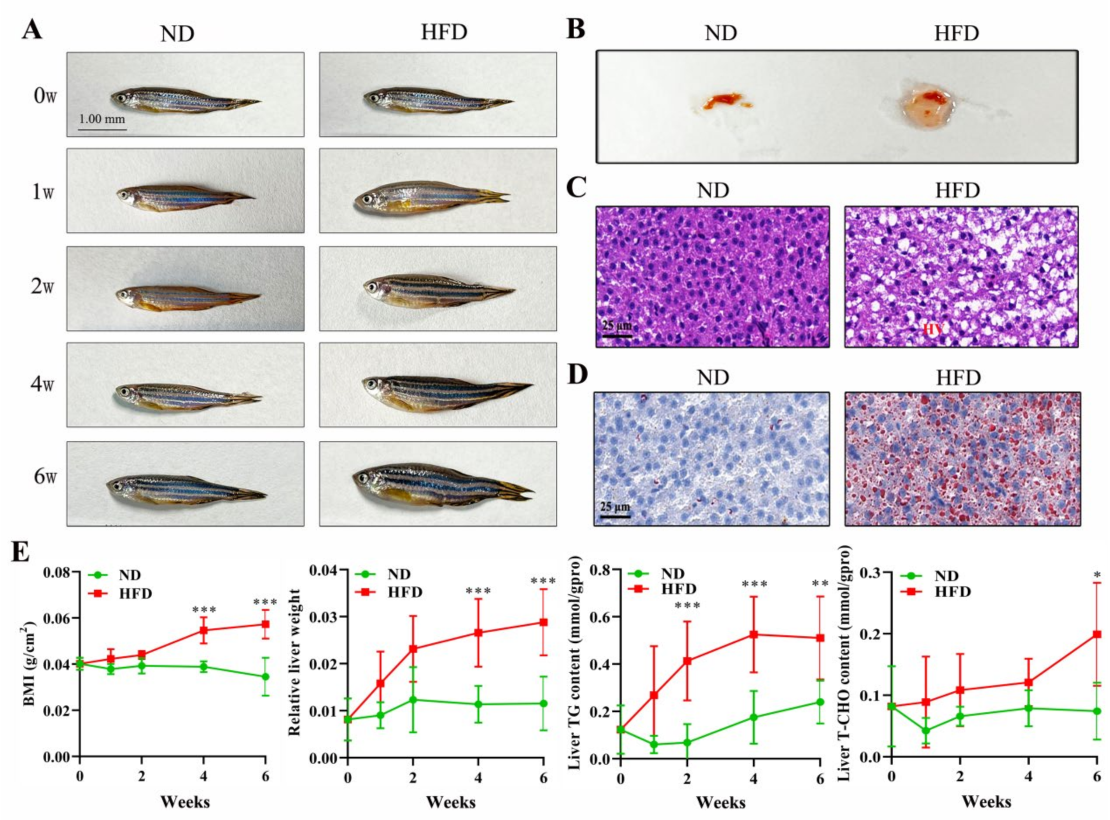

Supplement: Supplementary file 1 [file ijms-26-01360-s001.zip › ijms-3466465-supplementary.pdf]
